# Supplementary material for: Enhanced metanephric specification to functional proximal tubule enables toxicity screening and infectious disease modelling in kidney organoids
Source: Nat Commun. 2022 Oct 8;13:5943. doi: 10.1038/s41467-022-33623-z (PMC9547573; doi:10.1038/s41467-022-33623-z)
Supplement: Supplementary file 1 — Supplementary Information [file 41467_2022_33623_MOESM1_ESM.pdf]

Enhanced metanephric specification to functional proximal tubule enables toxicity screening and infectious disease modelling in kidney organoids

Jessica M. Vanslambrouck[1,2], Sean B. Wilson[1,2#], Ker Sin Tan[1#], Ella Groenewegen[1], Rajeev Rudraraju[3], Jessica Neil[3], Kynan T. Lawlor[1,2], Sophia Mah[1], Michelle Scurr[1], Sara E. Howden[1,2], Kanta Subbarao[3], Melissa H. Little[1,2,4*].

1. Murdoch Children's Research Institute, Flemington Rd, Parkville, VIC, Australia

2. Department of Paediatrics, The University of Melbourne, VIC, Australia.

3. Department of Microbiology and Immunology, The Peter Doherty Institute for Infection and Immunity, The University of Melbourne, VIC, Australia.

4. Department of Anatomy and Neuroscience, The University of Melbourne, VIC, Australia.

[#] Equal contribution

* Author for correspondence:

M.H.L.: +61 3 9936 6206; melissa.little@mcri.edu.au

Running title: Enhanced proximal tubules

Keywords: proximal tubule, pluripotent stem cell, kidney organoid, nephron patterning

Supplementary Figures and Legends

Supplementary Figure 1

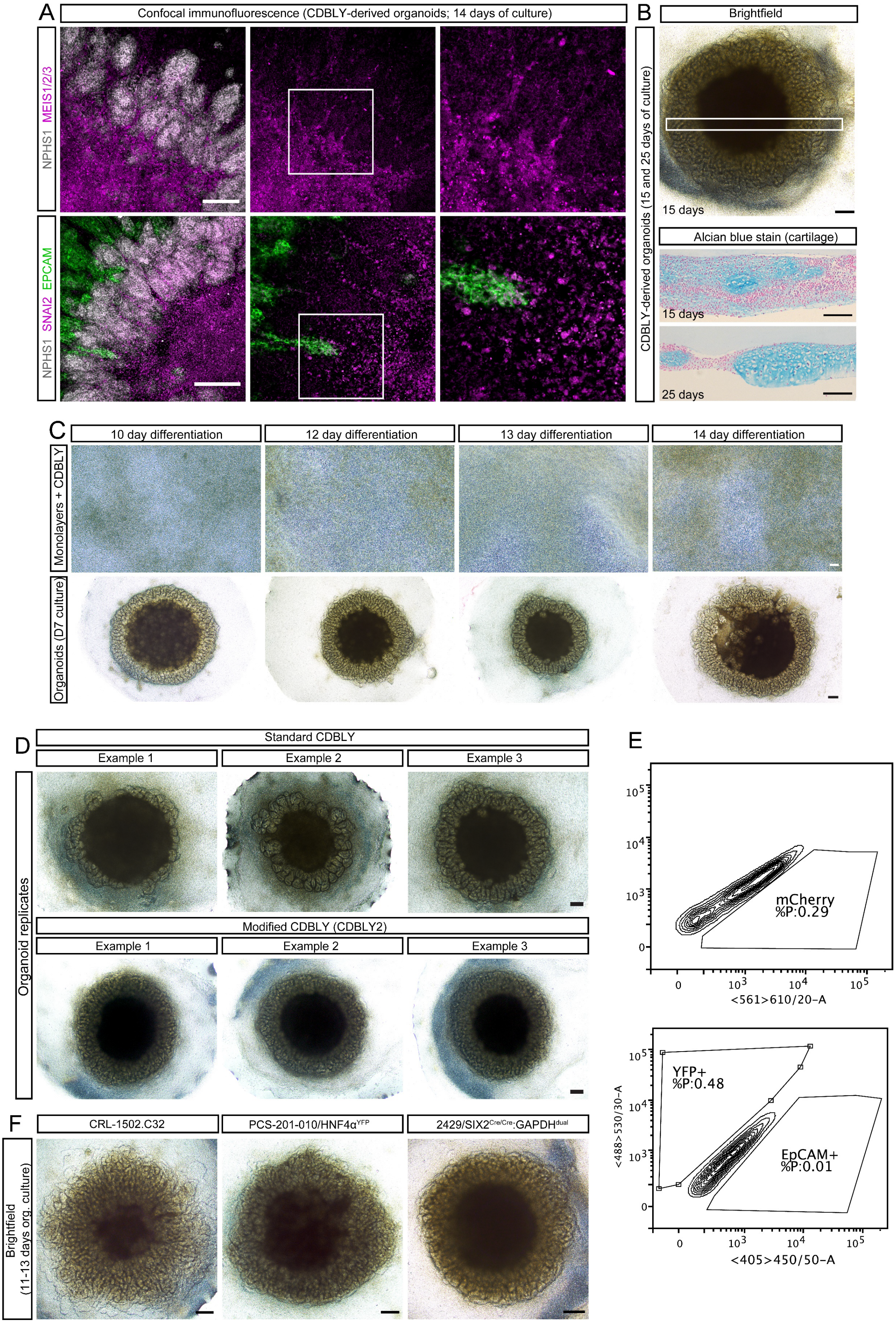

Supplementary Figure 1: Analyses of central core region and morphology of organoids resulting from extended monolayer differentiation in multiple cell lines. A. Confocal immunofluorescence of stromal markers, MEIS1/2/3 (magenta; top panels) and SNAI2 (magenta; bottom panels) in central core region of organoids derived from CDBLY-exposed extended monolayer differentiations. Organoids are co-stained for podocytes (NPHS1; grey) and epithelium (EPCAM; green). Scale bars represent 100 µm. **B.** Representative brightfield image (top) of a day 13+15 organoid exposed to CDBLY at monolayer differentiation day 8. White box indicates approximate regions of cross sections shown in bottom panels stained with Alcian blue, indicating patchy cartilage formation in central core region (blue). Scale bars represent 200 µm. **C.** Brightfield images of CDBLY-exposed monolayer differentiations extended for 10, 12, 13, and 14 days and their resulting organoids. Scale bars represent 100 µm (monolayers) and 200 µm (organoids). **D.** Brightfield images showing 3 examples of representative organoid morphologies derived from CDBLY-exposed (5 ng/mL BMP7) and CDBLY2-exposed (10 ng/mL BMP7) monolayer differentiations. Scale bars represent 200 µm. **E**. Flow cytometry plots exemplify the gating strategy for Figures 2B and 3B using dissociated kidney organoids that are unstained for EPCAM-488 and lack the reporters of interest (mCherry and YFP). FSC/SSC analysis was initially performed to identify individual live cells and exclude cell debris and clusters of two or more cells. **F.** Brightfield images of D13+11 – D13+13 organoids generated from multiple iPSC lines using extended monolayer differentiation with 5 days x 6µM CHIR exposure (CRL1502.C32; parental iPSC line derived from fetal female skin fibroblasts (Briggs*, et al.*, 2013), CRL-2429/SIX2$^{Cre/Cre}$:GAPDH^{dual}; lineage tracing reporter iPSC line originally derived from neonatal male foreskin fibroblasts (Howden*, et al.*, 2019; Vanslambrouck*, et al.*, 2019), and PCS-201-010/HNF4α^{YFP}; PT-specific fluorescence reporter iPSC line originally derived from neonatal male skin fibroblasts (Vanslambrouck*, et al.*, 2019). Scale bars represent 200 µm.

50 **Supplementary Figure 2**

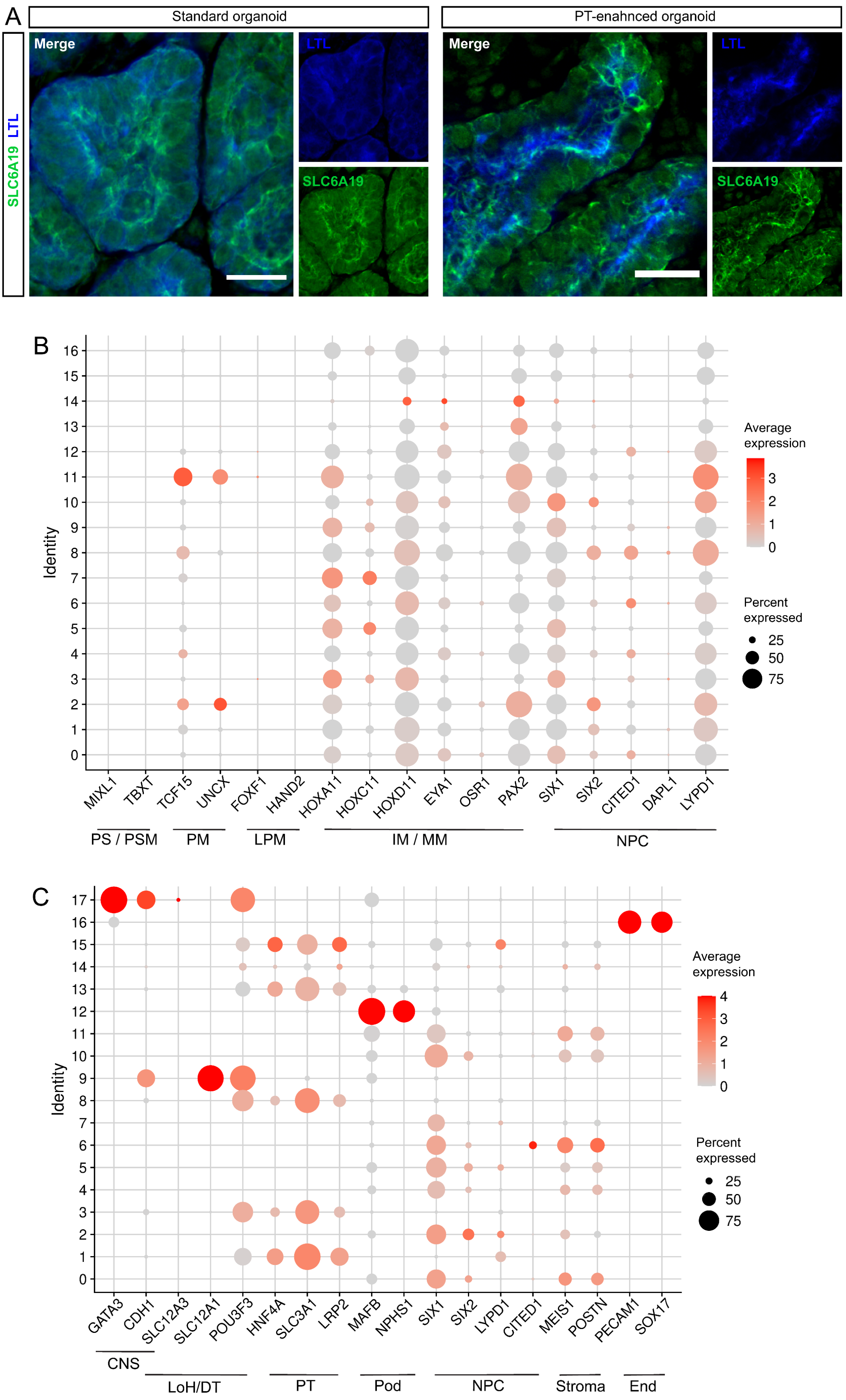

51

Supplementary Figure 2: Brush border membrane marker visualisation and scRNAseq cluster marker analyses of D13 monolayers and D13+14 PT-enhanced organoids. A. High-resolution confocal microscopy depicting immunofluorescence for apical PT brush border membrane marker LTL (blue) and SLC6A19 (green) in D7+14 (standard) and D13+14 (PT-enhanced) organoids. Scale bars represent 20µm. **B.** Dot plot of D13 combined replicate samples showing expression of early mesenchymal markers preceding metanephric kidney formation across all resolved clusters. Abbreviations: primitive streak (PS), presomitic mesoderm (PSM), paraxial mesoderm (PM), lateral plate mesoderm (LPM), intermediate mesoderm (IM), metanephric mesenchyme (MM), nephron progenitor cells (NPC). **C.** Dot plot of D13+14 combined replicate samples showing expression of kidney-specific markers across all resolved clusters. Abbreviations: connecting segments (CNS), loop of Henle (LoH), distal tubule (DT), proximal tubule (PT), podocyte (Pod), nephron progenitor cell (NPC), endothelium (End).

Supplementary Figure 3

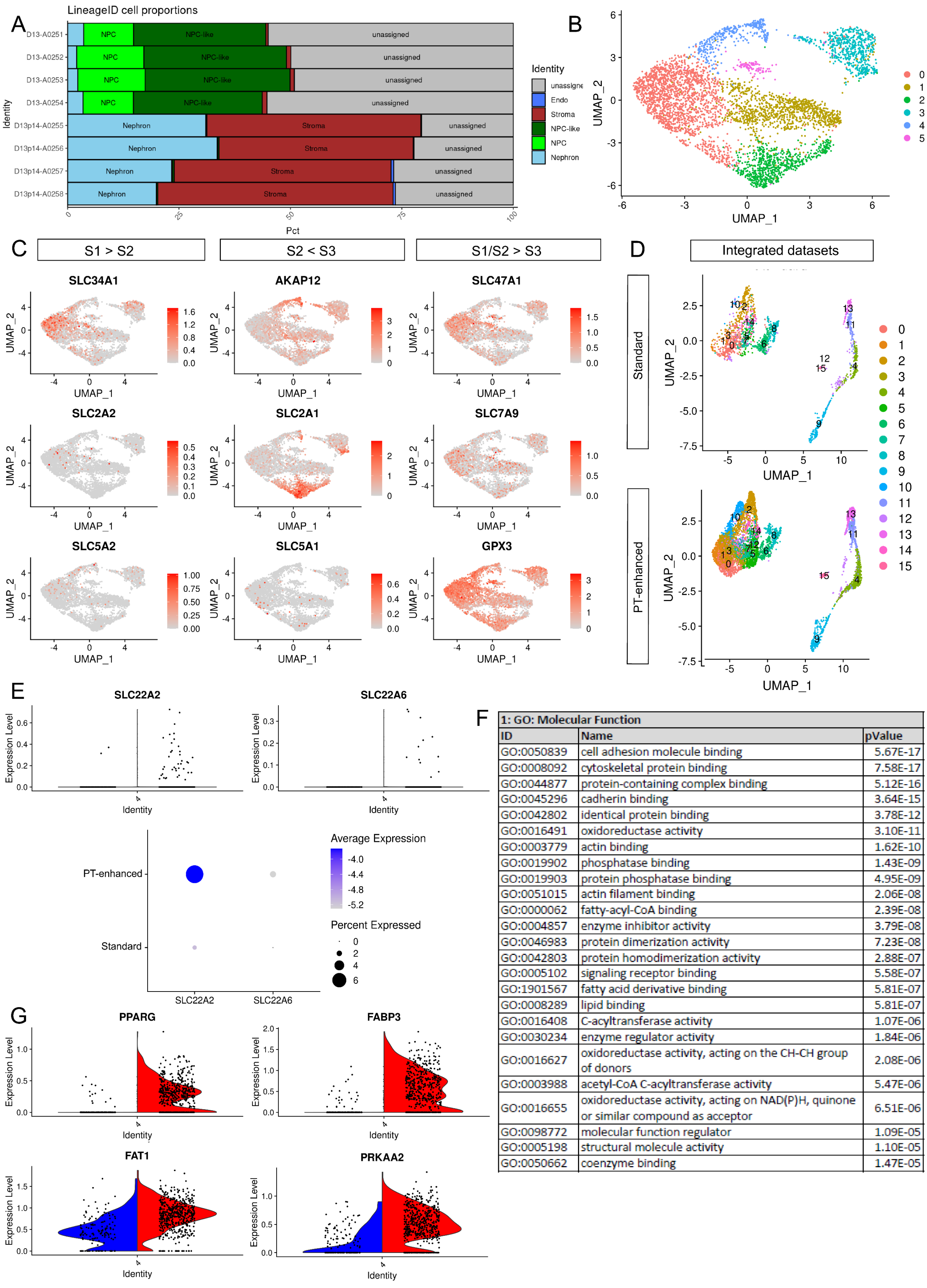

1: GO: Molecular Function		
ID	Name	pValue
GO:0050839	cell adhesion molecule binding	5.67E-17
GO:0008092	cytoskeletal protein binding	7.58E-17
GO:0044877	protein-containing complex binding	5.12E-16
GO:0045296	cadherin binding	3.64E-15
GO:0042802	identical protein binding	3.78E-12
GO:0016491	oxidoreductase activity	3.10E-11
GO:0003779	actin binding	1.62E-10
GO:0019902	phosphatase binding	1.43E-09
GO:0019903	protein phosphatase binding	4.95E-09
GO:0051015	actin filament binding	2.06E-08
GO:0000062	fatty-acyl-CoA binding	2.39E-08
GO:0004857	enzyme inhibitor activity	3.79E-08
GO:0046983	protein dimerization activity	7.23E-08
GO:0042803	protein homodimerization activity	2.88E-07
GO:0005102	signaling receptor binding	5.58E-07
GO:1901567	fatty acid derivative binding	5.81E-07
GO:0008289	lipid binding	5.81E-07
GO:0016408	C-acyltransferase activity	1.07E-06
GO:0030234	enzyme regulator activity	1.84E-06
GO:0016627	oxidoreductase activity, acting on the CH-CH group of donors	2.08E-06
GO:0003988	acetyl-CoA C-acyltransferase activity	5.47E-06
GO:0016655	oxidoreductase activity, acting on NAD(P)H, quinone or similar compound as acceptor	6.51E-06
GO:0098772	molecular function regulator	1.09E-05
GO:0005198	structural molecule activity	1.10E-05
GO:0050662	coenzyme binding	1.47E-05

Supplementary Figure 3: Comparison of D13 monolayers, PT-enhanced organoids, and published scRNAseq datasets. A. *ComparePlot* showing proportion of kidney cell types within each D13 and D13+14 replicate. **B.** UMAP plot of isolated PT clusters from D13+14 PT-enhanced organoids, re-clustered to resolve 6 distinct cell populations. **C.** UMAP plots showing the expression of S1, S2, and S3 segment markers within the isolated PT population of PT-enhanced D13+14 organoids. **D.** UMAP plots from integrated analyses of PT-enhanced organoids and our existing standard organoid dataset (Howden*, et al.*, 2019) (iPSC line- and age-matched). Clustering resolved 15 distinct cell populations for each sample in the integrated datasets. **E.** Violin (top panels) and dot plots (bottom panel) depicting *SLC22A2* and *SLC22A6* expression within the PT cluster of integrated PT-enhanced and standard organoid datasets from (**D**) (left and right on violin plots, respectively). **F.** Table depicting top 25 GO terms arising from unbiased ToppFun GO Molecular Function analyses of significantly differentially expressed genes between standard (blue, left) and PT-enhanced (red, right) organoid datasets from (**D**)**.** ToppGene P value method: Hypergeometric Probability Mass Function with FDR correction. **G.** Violin plots comparing examples of genes involved in fatty acid metabolism in standard (blue, left) and PT-enhanced (red, right) organoid datasets from **(D).**

Supplementary Figure 4

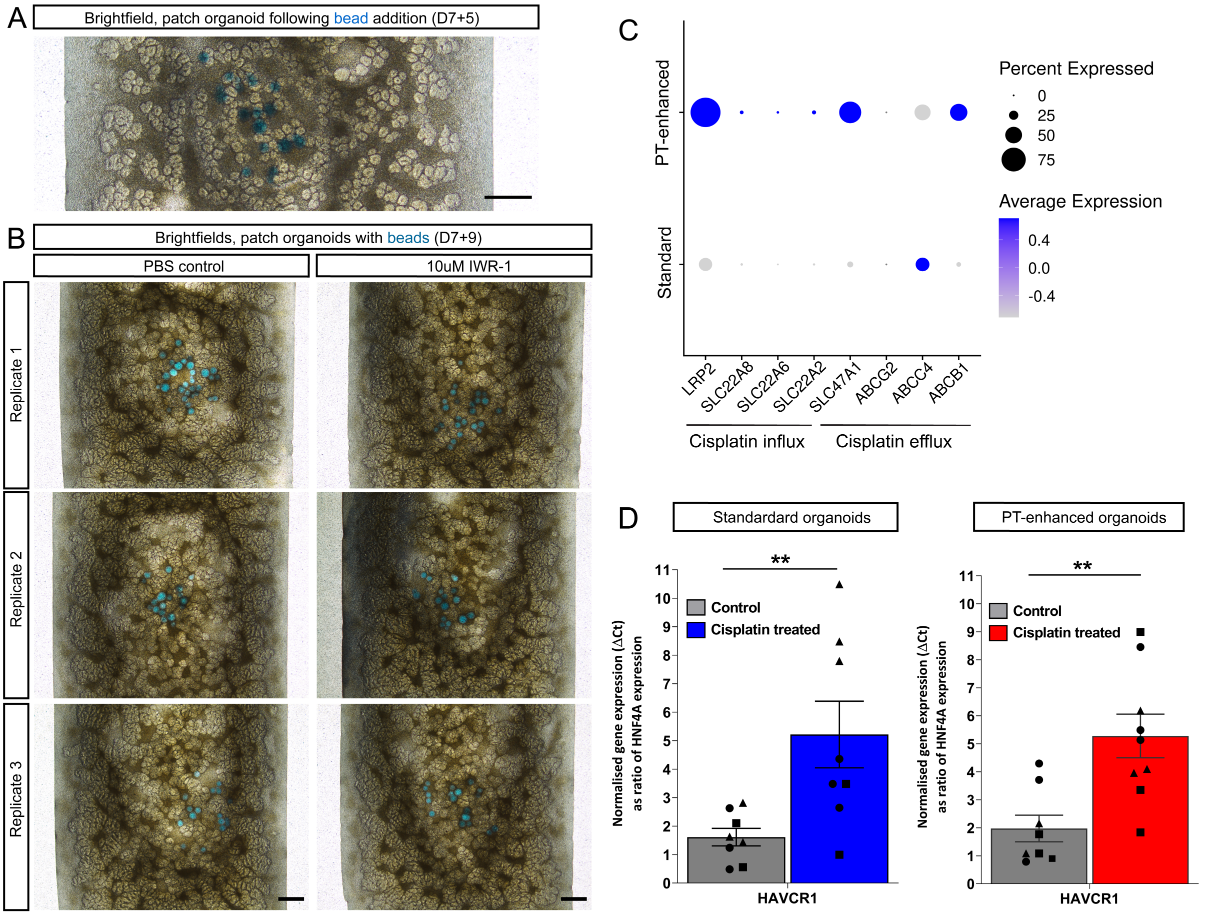

Supplementary Figure 4: Defining the mechanism of nephron directionality using IWR-1-soaked agarose beads and exploring PT-enhanced organoid functionality through cisplatin response. A. Brightfield image of a D7+5 standard bioprinted patch organoid immediately after the addition of agarose beads (blue) depicting their contact with forming renal vesicle structures. Scale bar represents 200 μm. **C.** Brightfield images of D7+9 bioprinted patch organoids containing PSB-soaked or IWR1-soaked (left and right panels, respectively) beads (blue). Scale bars represent 200μm. **C.** scRNAseq dot plot comparing the expression of cisplatin influx and efflux transporters within the PT cluster of integrated PT-enhanced and existing standard organoid datasets (Howden, *et al.*, 2019) (iPSC line- and age-matched). **D.** qRT-PCR analyses depicting KIM-1 gene (*HAVCR1*) expression in standard (blue; left panel) and PT-enhanced (red; right panel) organoids treated with cisplatin, compared to their respective PBS-treated controls (grey). *HAVCR1* gene expression values are normalised to the housekeeping gene *GAPDH* ($2^{-\Delta Ct}$) and expressed as a ratio of *HNF4A* to compensate for differences in proximal tubule proportion. Error bars represent SEM from n = 8 (control) and n = 9 (cisplatin-treated) biological replicates across 3 replicate experiments as indicated.

Statistical significance was determined using an unpaired t test. Asterisks represent two-tailed P values (**; $P \leq 0.01$ [Standard organoids: $P = 0.009858$; PT-enhanced organoids: $P = 0.003110$]]) adjusted for multiple comparisons using the Holm-Sidak method alpha = 0.05. Source data are provided as a Source Data file.

Supplementary Figure 5

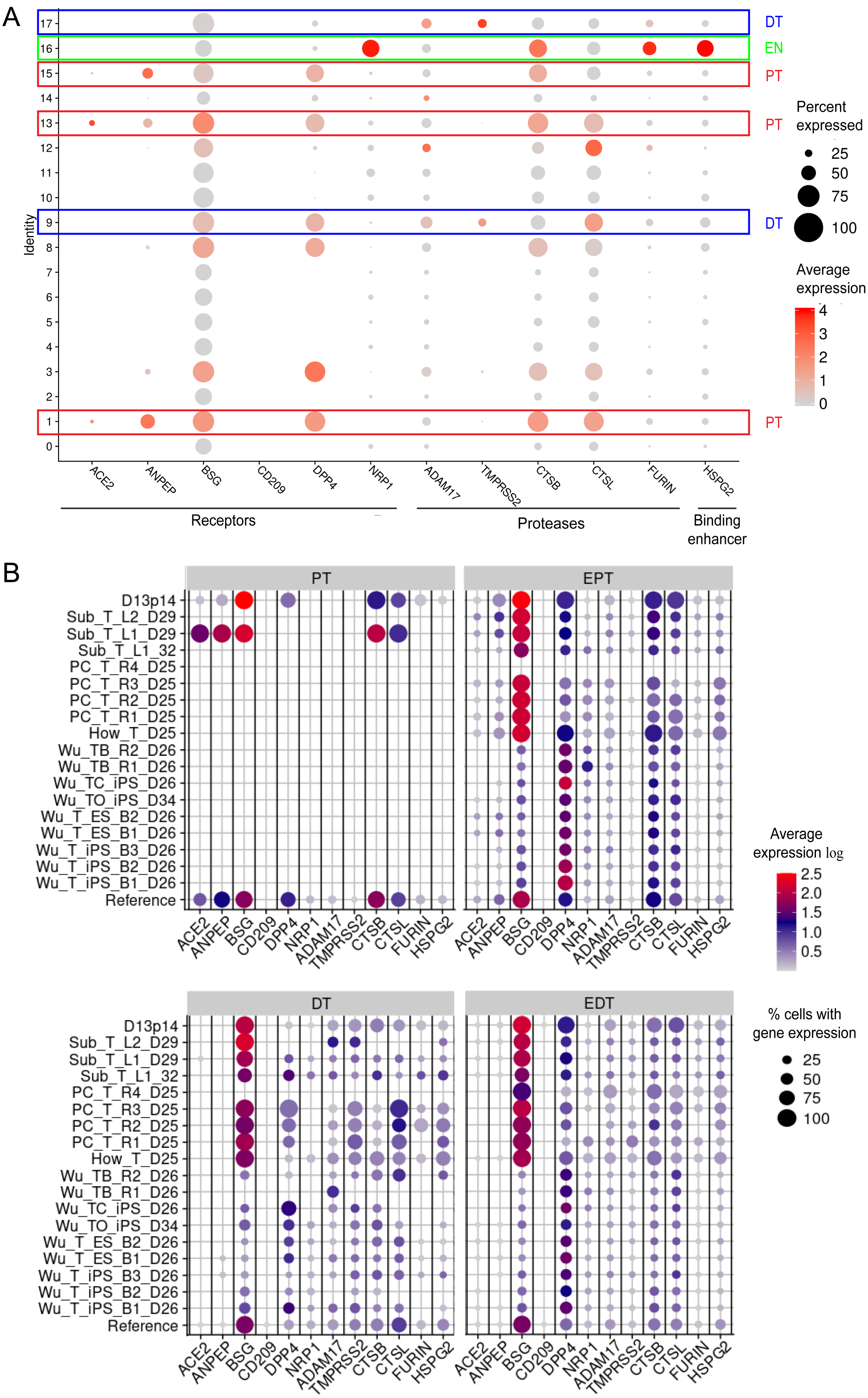

Supplementary Figure 5: Expression of SARS-CoV-2 entry factors kidney organoids. A. scRNAseq analysis of SARS-CoV-2 entry factor expression in PT-enhanced (D13+14) kidney organoids. Boxes outline proximal (red), distal (blue), and endothelial (green) clusters. **B.** *DotPlotCompare* generated using the *DevKidCC* package (Wilson*, et al.*, 2022) showing the comparison of SARS-CoV-2 entry factor expression between PT-enhanced organoids (D13p14), stem cell-derived kidney organoid datasets (Subramanian*, et al.*, 2019; Wu*, et al.*, 2018; Howden*, et al.*, 2019; Combes*, et al.*, 2019; Phipson*, et al.*, 2019), and the mixed week 11 - 18 fetal kidney reference dataset (Hochane*, et al.*, 2019; Tran*, et al.*, 2019; Hollywood*, et al.*, 2020) within populations classified as proximal tubule (PT), early proximal tubule (EPT), distal tubule (DT), and early distal tubule (EDT). Dot colour and size represents unscaled gene expression and percentage of cells expressing each gene, respectively.

Supplementary Figure 6

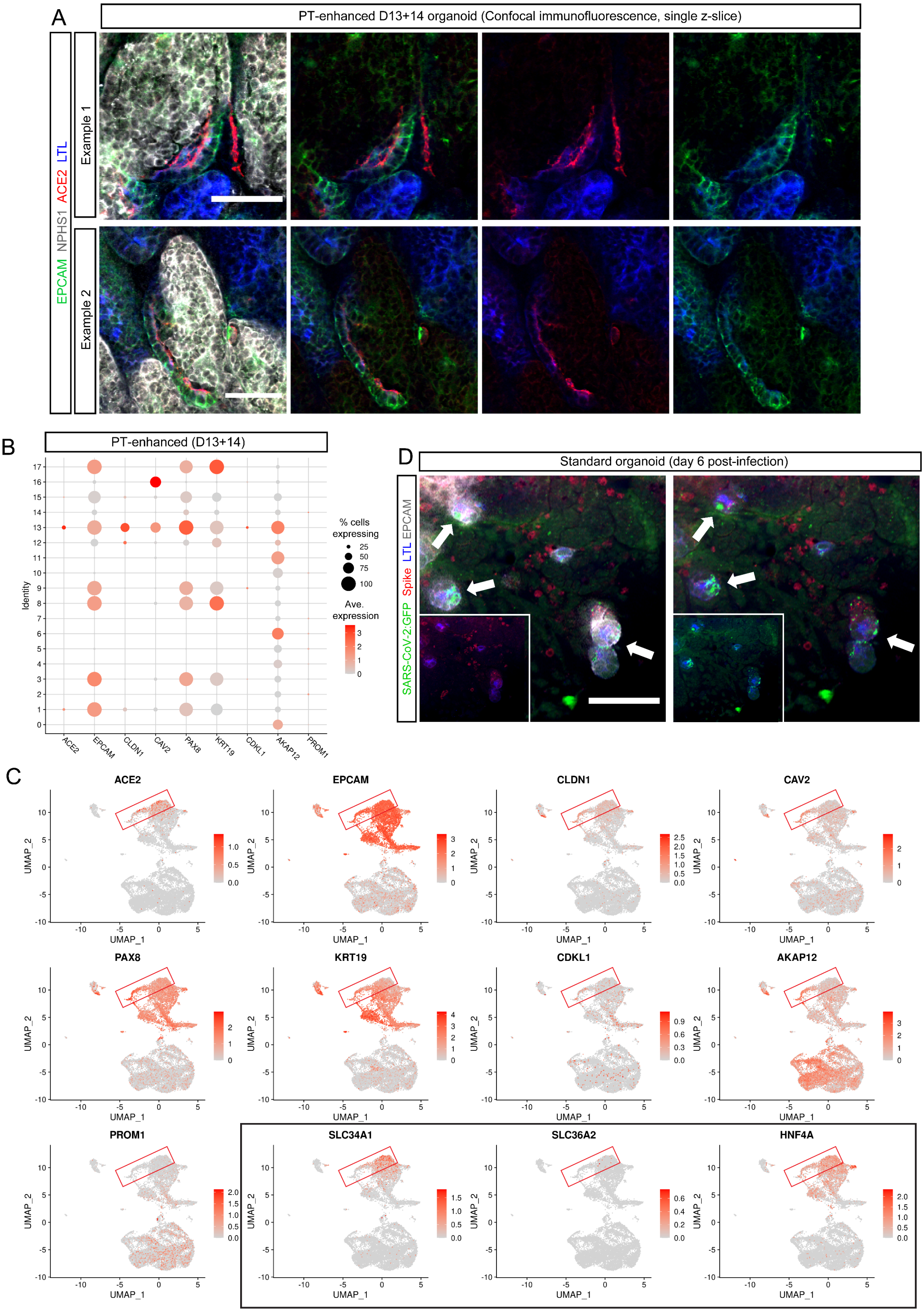

Supplementary Figure 6: Expression of SARS-CoV-2 entry factors in kidney organoid PEC populations and confirmation of infectious virus presence. A. Confocal immunofluorescence of a D13+14 PT-enhanced organoid depicting apical ACE2 (red) expression on EPCAM-positive (green) cells entering the early portion of Bowman's capsule surrounding NPHS1-positive (grey) podocytes of glomeruli. LTL (blue) marks PT. Scale bars represent 50µm. **B-C.** Analyses of the PT-enhanced D13+14 scRNAseq dataset displayed as a dot plot (**B**) and feature plots (**C**), depicting expression of *ACE2* in clusters co-expressing markers of cuboidal and intermediate PECs, as well S1-specific markers of PT. Boxes in (**C**) highlight the key region of overlapping expression in the PT clusters. **D.** Confocal immunofluorescence of standard (D7+20) organoid 6 days post-infection with GFP-tagged SARS-CoV-2 confirming the presence of infectious viral particles (GFP-positive [green] and spike protein-expressing [red]), showing evidence of mature and replicating virus within $EPCAM^+$/LTL positive (blue) PTs as well as the interstitium. Insets depict 2-channel overlays of larger merged images. Arrows indicate examples of viral GFP in LTL-positive tubules. Scale bar represents 50µm.

Supplementary Figure 7

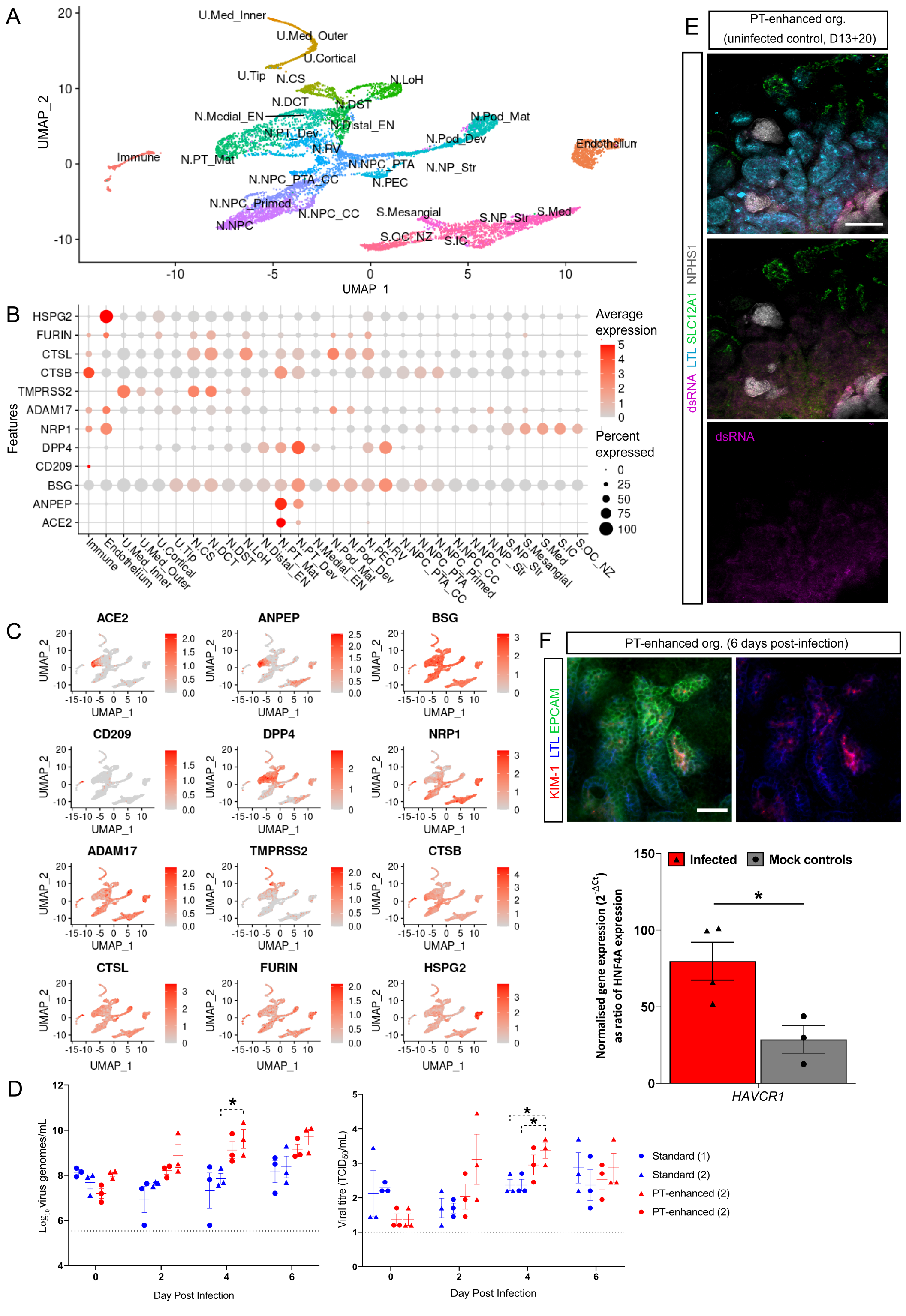

Supplementary Figure 7: SARS-CoV-2 entry factor distribution in human fetal kidney and infectivity of PT-enhanced kidney organoids. **A-C.** Single cell RNAseq analysis of week 11 – 18 mixed human fetal kidney reference datasets (Hochane*, et al.*, 2019; Tran*, et al.*, 2019; Holloway*, et al.*, 2020) displayed in UMAP (**A** and **C**) and dot plot (**B**) formats confirming the resolution of kidney cell clusters and SARS-CoV-2 entry factor expression. Cluster abbreviations: ureteric (U), medullary (Med), nephron (N), connecting segment (CS), distal convoluted tubule (DCT), distal straight tubule (DST), loop of Henle (LoH), early nephron (EN), proximal tubule (PT), developing (Dev), maturing (Mat), podocyte (Pod), parietal epithelial cell (PEC), renal vesicle (RV), nephron progenitor cell (NPC), pre-tubular aggregate (PTA), cycling cells (CC), nephron progenitor (NP), stroma (S), stromal (Str), inner cortical (IC), outer cortical (OC), nephrogenic zone (NZ). **D.** Viral titre data from Figure 7C re-displayed as scatter plots (left panel: qRT-PCR of *E* gene levels; right panel: $TCID_{50}$ readings), depicting representative experiments ([1] and [2]) using standard (blue) and PT-enhanced (red) conditions. Dotted line represents lower limit of detection and mock-infected control readings. Error bars indicate mean and SEM (n = 3 individual wells; 3 organoids per well per timepoint). Statistical significance was determined using a one-way ANOVA with Tukey's multiple comparisons test. Asterisks represent two-tailed P values (*; $P \leq 0.05$, P = 0.0297 [left plot] and 0.0457 [right plot]) Source data are provided as a Source Data file. **E.** Confocal immunofluorescence of a representative matched control organoid for SARS-CoV-2 infection experiments (uninfected PT-enhanced D13+20 organoid), stained for viral RNA (dsRNA; magenta), PT (LTL; cyan), LoH (SLC12A1; green), and podocytes (NPHS1; grey). Scale bar represents 100µm. **F.** Top panel depicts confocal immunofluorescence of a PT-enhanced organoid 6 day- post infection, showing KIM-1 (red) within EPCAM-positive/LTL-positive (green/blue) PTs. Scale bar represents 50µm. Bottom panel depicts qRT-PCR of KIM-1 gene (*HAVCR1*) expression in infected (red) and uninfected control (grey) PT-enhanced organoids 6 days post-infection. *HAVCR1* values are normalised to *GAPDH* and expressed as a ratio of *HNF4A* (compensating for PT proportion variation). Error bars represent SEM (n = 4 (infected) and n = 3 (mock) biological replicate organoids. Asterisk (*) represents P value ≤ 0.05 (P = 0.0267). Statistical significance was determined using an unpaired t test adjusted for multiple comparisons using the Holm-Sidak method with alpha = 0.05.

Supplementary Tables

sample	condition	bead_radius	gloms_in_radius	ltl_in_radius	epcam_in_radius	tissue_in_radius
1	IWR	1905017	495180	41898	92217	577495
2	IWR	1436790	592680	27171	69770	621571
3	IWR	1732366	746348	40231	99303	838595
1	PBS	1995803	722679	115372	241557	946907
2	PBS	1404122	651280	78934	203536	825990
3	PBS	1877655	734616	72331	204901	916286

Supplementary Table 1: Quantification of nephron structures in organoids exposed to IWR1-soaked and PBS-soaked agarose beads (bead_radius: total number of pixels that are within 200 pixels from a bead edge; gloms_in_radius: total number of pixels defined as NPHS1 positive, within 200 pixels from a bead edge; ltl_in_radius: total number of pixels defined as LTL positive, within 200 pixels from a bead edge; epcam_in_radius: total number of pixels defined as EPCAM positive, within 200 pixels from a bead edge; tissue_in_radius: sum of total number of pixels defined as NPHS1 or EPCAM positive, within 200 pixels from a bead edge).
