# Supplementary material for: Enhanced metanephric specification to functional proximal tubule enables toxicity screening and infectious disease modelling in kidney organoids
Source: Nat Commun. 2022 Oct 8;13:5943. doi: 10.1038/s41467-022-33623-z (PMC9547573; doi:10.1038/s41467-022-33623-z)
Supplement: Supplementary file 2 — Description of Additional Supplementary Files [file 41467_2022_33623_MOESM2_ESM.pdf]

Description of Additional Supplementary Files

Supplementary Data 1: Differentially expressed (DE) genes by cluster in day 13 (D13) monolayers derived from extended differentiation in CDBLY2. Differential expression was calculated using a two-tailed non-parametric Wilcoxon rank sum test. pVal represents adjusted P values based on Bonferroni correction using the total number of genes in the dataset.

Supplementary Data 2: Differentially expressed (DE) genes by cluster in D13+14 organoids derived from D13 monolayers Differential expression was calculated using a two-tailed non-parametric Wilcoxon rank sum test. pVal represents adjusted two-sided P values based on Bonferroni correction using the total number of genes in the dataset.
