# Supplementary material for: Enhanced metanephric specification to functional proximal tubule enables toxicity screening and infectious disease modelling in kidney organoids
Source: Nat Commun. 2022 Oct 8;13:5943. doi: 10.1038/s41467-022-33623-z (PMC9547573; doi:10.1038/s41467-022-33623-z)
Supplement: Supplementary file 5 — Reporting Summary [file 41467_2022_33623_MOESM5_ESM.pdf]

nature portfolio

Corresponding author(s): Melissa H Little

Last updated by author(s): Sep 1, 2022

Reporting Summary

Nature Portfolio wishes to improve the reproducibility of the work that we publish. This form provides structure for consistency and transparency in reporting. For further information on Nature Portfolio policies, see our Editorial Policies and the Editorial Policy Checklist.

Statistics

For all statistical analyses, confirm that the following items are present in the figure legend, table legend, main text, or Methods section.

n/a	Confirmed	
☐	☒	The exact sample size (*n*) for each experimental group/condition, given as a discrete number and unit of measurement
☐	☒	A statement on whether measurements were taken from distinct samples or whether the same sample was measured repeatedly
☐	☒	The statistical test(s) used AND whether they are one- or two-sided<br>*Only common tests should be described solely by name; describe more complex techniques in the Methods section.*
☒	☐	A description of all covariates tested
☐	☒	A description of any assumptions or corrections, such as tests of normality and adjustment for multiple comparisons
☐	☒	A full description of the statistical parameters including central tendency (e.g. means) or other basic estimates (e.g. regression coefficient) AND variation (e.g. standard deviation) or associated estimates of uncertainty (e.g. confidence intervals)
☐	☒	For null hypothesis testing, the test statistic (e.g. *F*, *t*, *r*) with confidence intervals, effect sizes, degrees of freedom and *P* value noted<br>*Give P values as exact values whenever suitable.*
☒	☐	For Bayesian analysis, information on the choice of priors and Markov chain Monte Carlo settings
☒	☐	For hierarchical and complex designs, identification of the appropriate level for tests and full reporting of outcomes
☒	☐	Estimates of effect sizes (e.g. Cohen's *d*, Pearson's *r*), indicating how they were calculated

Our web collection on statistics for biologists contains articles on many of the points above.

Software and code

Policy information about availability of computer code

Data collection	Imaging data were collected using ZEISS ZEN 2012 (blue edition) or ZEISS ZEN Black software (version 2.3 SP1) (Zeiss Microscopy, Thornwood, NY) software installed on a ZEISS LSM 780 confocal microscope or Zeiss Axio Imager A2 (Carl Zeiss, Oberkochen, Germany). qRT-PCR data were collected using the Applied Biosystems 7500 Sequence Detection Software (version 1.5.1) installed on the Applied Biosystems 7500 Real Time PCR System. Single cell RNA sequencing data were collected with NovaSeq Control Software for the Illumina NovaSeq 6000 sequencing system. Flow cytometry data were collected using the BD LSR Fortessa X-20 Cell Analyser (BD Biosciences, CA).
Data analysis	Image processing was performed using ZEISS ZEN Black software (version 2.3 SP1) (Zeiss Microscopy, Thornwood, NY), Fiji ImageJ (version: 2.1.0/1.53c) (Schindelin, et al., 2012), or Python (version 3.10.2) libraries as described in the Methods, including image processing, pixel distance calculations, and data display using Numpy (version 1.22.1), Czifile library (version 2019.7.2), Napari (version 0.4.13), Scikit-image (version 0.19.2), Euclidian distance transform in Scipy (version 1.7.3) (Virtanen, et al., 2020), and Matplotlib (version 3.5.1). Quantitative RT-PCR (qRT-PCR) data and viral quantification data were graphed and analysed in GraphPad Prism (version 8). Flow cytometry data were analysed using FACsDiva versions 8.0.1 and 9.0.1 (BD Biosciences) and FlowLogic software version 8.6 (Invai). Single cell RNA sequencing (scRNASeq) data were demultiplexed using CellRanger (version 3.1.0) (10x mRNA libraries) and Cite-seq-count (version 1.4.3) (HTO libraries) before loading into Seurat (version 3.1.4) for downstream analyses where data were normalised using the SCTransform method (Hafemeister and Satija, 2019) and marker analysis was performed using the Seurat FindMarkers function. Exported scRNAseq marker lists were subjected to gene ontology analysis usingToppFun (version: 2021-Mar-29 15:59 / #31 / 639dfe36cbad55ba69e8255099537d5c2fcb9483) (https://toppgene.cchmc.org/enrichment.jsp). Comparative analyses of D13 and D13+14 datasets with existing published datasets were performed using DevKidCC (version 0.3.0) (Wilson SB. et al., 2021) and the DotPlotCompare function. Custom codes are available as stated in the manuscript Code Availability section and as follows: Code for scRNAseq and image analyses are available through the Github repository (https://github.com/KidneyRegeneration/Vanslambrouck2022; DOI: 10.5281/zenodo.7021393) (Vanslambrouck, et al., 2022).

For manuscripts utilizing custom algorithms or software that are central to the research but not yet described in published literature, software must be made available to editors and reviewers. We strongly encourage code deposition in a community repository (e.g. GitHub). See the Nature Portfolio guidelines for submitting code & software for further information.

Data

Policy information about availability of data

All manuscripts must include a data availability statement. This statement should provide the following information, where applicable:

- Accession codes, unique identifiers, or web links for publicly available datasets
- A description of any restrictions on data availability
- For clinical datasets or third party data, please ensure that the statement adheres to our policy

The transcriptional profiling datasets generated in this study have been deposited in GEO under accession code GSE184928 [https://www.ncbi.nlm.nih.gov/geo/query/acc.cgi?acc=GSE184928]. The raw data from scRNAseq and immunofluorescence image analyses have been deposited in the Github repository [https://github.com/KidneyRegeneration/Vanslambrouck2022]. Raw and processed data from qRT-PCR, TCID50 assays, and image analyses are provided in the Source Data file.

Field-specific reporting

Please select the one below that is the best fit for your research. If you are not sure, read the appropriate sections before making your selection.

- [x] Life sciences
- [ ] Behavioural & social sciences
- [ ] Ecological, evolutionary & environmental sciences

For a reference copy of the document with all sections, see nature.com/documents/nr-reporting-summary-flat.pdf

Life sciences study design

All studies must disclose on these points even when the disclosure is negative.

Sample size

Sample sizes were based on even group distribution and no statistical method was used to predetermine sample size owing to the exploratory nature of the study. Experiments included a minimum of 3 biological replicates per condition across multiple experiments. Biological replicates were classed as monolayer differentiations or organoids derived from separate wells.

Data exclusions

Minimal data were excluded from the study. Organoids that failed to form kidney structures were omitted from downstream analyses without affecting sample size. For scRNASeq studies, cells were excluded based on poor QC scores and barcode readouts that indicate empty cells or multiplets. For qRT-PCR experiments, a technical replicate or sample was excluded if amplification failed. For SARS-CoV-2 infection experiments, one experimental replicate was excluded from downstream analyses due to a failure to infect.

Replication

The results depicted in this manuscript are representative of the similar observations and analyses made across multiple independent experiments, biological replicates, and technical replicates. Each experiment included a minimum of 3 biological replicates per condition across multiple experiments. Biological replicates were classed as monolayer differentiations or organoids derived from separate wells. To ensure robustness and reproducibility, experiments were replicated multiple times as either stated in the Figure Legend or as follows. Differentiation condition comparisons (Figure 1BC, Supplementary Figure 1CD) using CDBLY and standard E6 conditions were replicated in more than 5 independent experiments, including NPSR and CDBLY modifications (exposure and composition) in 2 independent experiments, with between 3 and 8 organoids generated per condition (depending on monolayer cell counts). Successful generation of organoids from multiple iPSC lines using the extended differentiation protocol (Supplementary Figure 1F) was replicated in more than 5 independent experiments, with more variability in morphology notable in PCS-201-010/HNF4αYFP. Lineage tracing of SIX2-mCherry cells in PT-enhanced organoids (Figure 2AB) was performed across 4 independent experiments of 3 or 4 biological replicates. Observations of radially aligned and proximilised nephrons in PT-enhanced organoids compared to standard organoids (Figure 2CD) have been made across more than 10 independent experiments. Improved PT marker protein expression in PT-enhanced organoids compared to standard organoids (Figure 3) has been assessed and observed in 3 independent experiments, with stromal marker analyses (Supplementary Figure 1A) performed in 3 organoids matched to one these same experiments. Alcian Blue cartilage staining (Supplementary Figure 1B) was performed and similarly detected in 4 separate PT-enhanced organoids across 3 independent experiments. PT brush border membrane characteristics (Supplementary Figure 2A) of PT-enhanced organoids compared to standard organoids were replicated in more than 3 independent experiments, with apical SLC6A19 expression observed in standard organoids derived from 1 out of 3 independent experiments. Fluorescent substrate uptake assays for PT-enhanced and standard organoids (Figure 6A) included 3 individual wells of 3 organoids per substrate/control condition and uptake was assessed in 4 independent experiments (2 per organoid protocol) with identical results. IWR-1 soaked agarose beads (Supplementary Figure 4AB) were added to organoids in 4 independent experiments, with influences on organoid morphology observed in 3 of these experiments (1 independent experiment excluded from downstream analyses owing to failed organoid differentiation). SARS-CoV-2 entry factor detection via immunofluorescence (Figure 7B, Supplementary Figure 6A) has been replicated in 3 independent experiments. Viral protein detection (Figure 7C, Supplementary Figure 6D) has been performed in 3 independent experiments with variation in viral protein intensity and abundance owing to variable infection levels between individual organoids and organoid batches.

Randomization

Assortment of well-formed organoids of equivalent morphology into experimental groups was randomized for all experiments. Each experiment included a minimum of 3 biological replicates per condition across multiple experiments. Biological replicates were classed as monolayer differentiations or organoids derived from separate wells.

Blinding

Investigators were not blinded to allocation during analyses and outcome assessment owing to the automated processes used to collect and analyse results.

March 2021

Reporting for specific materials, systems and methods

We require information from authors about some types of materials, experimental systems and methods used in many studies. Here, indicate whether each material, system or method listed is relevant to your study. If you are not sure if a list item applies to your research, read the appropriate section before selecting a response.

Materials & experimental systems

n/a	Involved in the study
☐	☒ Antibodies
☐	☒ Eukaryotic cell lines
☒	☐ Palaeontology and archaeology
☒	☐ Animals and other organisms
☒	☐ Human research participants
☒	☐ Clinical data
☒	☐ Dual use research of concern

Methods

n/a	Involved in the study
☒	☐ ChIP-seq
☐	☒ Flow cytometry
☒	☐ MRI-based neuroimaging

Antibodies

Antibodies used

All antibodies used are detailed in Table 1, detailed below:

ACE2 Rabbit polyclonal IgG 1:300 (Abcam (ab15348) Lot GR3333640-R)

CUBILIN (Y-20) Goat polyclonal IgG 1:300 (Santa Cruz Biotechnology (sc-20607) Lot J1012)

dsRNA Mouse monoclonal IgG2a, Kappa 1:300 (Absolute Antibody (Ab01299-2.0) clone J2, Lot T2007A26)

ECADHERIN Mouse monoclonal IgG2a, Kappa 1:300 (BD Biosciences (610181) clone 36)

EpCAM (Alexa488 or Alexa647 conjugate) Mouse monoclonal IgG2a, Kappa 1:300 (BioLegend (324210 and 324212) clone 9C4, Lot B352438 and B314235)

GATA3 Goat polyclonal IgG or rabbit monoclonal IgG 1:300 (R&D Systems (AF2605) and Cell Signalling Technology (5852S clone D13C9. Lot UZQ0219011))

GFP Chicken polyclonal IgY 1:200 - 1:300 (Abcam (ab13970) Lot GR3361051-11)

HNF4A Mouse monoclonal IgG2a 1:300 (Invitrogen (MA1-199) clone K9218, Lot WI3366551)

KIM-1 Goat polyclonal IgG 1:300 (R&D Systems (AF1750), Lot JTB0821051)

mCherry (RFP) Rabbit polyclonal IgG 1:300 – 1:400 (MBL Medical & Biological Laboratories Co. Ltd. (PM005), Lot 048)

MEGALIN Rabbit polyclonal IgG 1:300 (Novus Biologicals (NBP2-39033), Lot R91329)

NEPHRIN Sheep polyclonal IgG 1:300 (R&D Systems (AF4269), Lot ZMU0221031)

Proximal tubule brush border membrane Lotus tetragonobulus lectin (LTL), biotinylated 1:300 – 1:500 (Vector Laboratories (B-1325-2))

TMPRSS2 Mouse monoclonal IgG1, Kappa 1:300 (Merck (MABF2158) clone P5H9-A3, Lot 351024)

TotalSeq-A anti-human hashtag oligo antibody, 1:50 (BioLegend TotalSeq-A0251 to A0258)

S2 subunit of SARS-CoV-2 spike protein (stain: Sin2774) Rabbit monoclonal IgG1 1:300 (GeneTex (GTX632604) clone HL1002, Lot 44405)

SLC6A19 Chicken 1:100 – 1:200 (Aves Laboratories (custom antibody))

SLC12A1 Rabbit polyclonal IgG 1:300 – 1:400 Proteintech (18970-1-AP), Lot 00045810

Validation

Antibodies were previously validated for immunofluorescence of human or mouse tissue either in the laboratories of ourselves or commercial suppliers. Where necessary, antibody specificity was tested with pre-blocking of corresponding peptide.

Eukaryotic cell lines

Policy information about cell lines

Cell line source(s)	Non-reporter iPSC lines used in this study include CRL1502.C32 (cell line derived from WS1 CRL-1502TM female fibroblasts from ATCC) (Takasato, et al., 2015; Briggs, et al., 2013) and PB010/MCRIi010-A (derived from peripheral blood mononuclear cells of healthy adult individuals (Vlahos, et al., 2019)). Reporter iPSC lines used in this study include CRL-2429/SIX2Cre/Cre:GAPDHdual and PCS-201-010/HNF4AYFP (derived from human foreskin fibroblasts [CCD-1112Sk/CRL-2429TM, ATCC] and primary dermal fibroblasts [PCS-201-101TM, ATCC], respectively, with simultaneous reprogramming and reporter line generation performed in-house. Reporter lines are available for distribution from the Washington University Kidney Translational Research Centre, St Louise, MO, accessible via the ReBuilding a Kidney Reporter Cell Line database [https://www.rebuildingakidney.org/chaise/recordset/#2/Cell_Line:Reporter_Cell_Line@sort(RID] and identified as "SIX2:Cre/GAPDH:Dual" and "HNF4A:YFP", respectively) (Vanslambrouck, et al., 2019; Howden, et al., 2019). Vero cells were obtained from ATCC (#CCL-81).
Authentication	iPSC lines are checked for pluripotency and genomic integrity by immunofluorescence of pluripotency markers, molecular karyotyping and G-Banding. Vero cells purchased through ATCC were not authenticated
Mycoplasma contamination	iPSC lines and Vero cells tested negative for mycoplasma contamination.
Commonly misidentified lines (See ICLAC register)	No commonly misidentified cell lines were used.

Flow Cytometry

Plots

Confirm that:

- [x] The axis labels state the marker and fluorochrome used (e.g. CD4-FITC).
- [x] The axis scales are clearly visible. Include numbers along axes only for bottom left plot of group (a 'group' is an analysis of identical markers).
- [x] All plots are contour plots with outliers or pseudocolor plots.
- [x] A numerical value for number of cells or percentage (with statistics) is provided.

Methodology

Sample preparation	Flow cytometry of reporter line-derived organoids using endogenous fluorescence was performed and analysed following published methodology and as follows (Vanslambrouck, et al., 2019). Following Accutase (StemCell Technologies) dissociation of the organoids at 37ºC (1mL Accutase per 6 organoids for 15 minutes, pipetting every 3 minutes), the enzymes were inactivated with TeSR-E6 / 2% FBS and by placing the cell suspension on ice for 2 minutes. Dissociated organoid cells were passed through 40µm and 70µm cell strainers with additional TeSR-E6 / 2% FBS prior to centrifugation (1500rpm for 3 minutes) and resuspension in 100 – 500µL PBS containing 1% FBS (FACS wash). To determine the contribution of SIX2-mCherry + cells to EPCAM+ populations in organoids derived from the SIX2Cre lineage tracing iPSC line, cells were stained using directly conjugated anti-EPCAM Alexa Fluor-647 antibody (see Table 1) diluted 1:100 in 100 µL of FACS wash for every 5 x105 cells. Following 30 minutes incubation on ice, stained cells were washed 3 times in 2mL FACS wash via centrifugation prior to flow cytometry.
Instrument	Flow cytometry was performed using the BD LSR Fortessa X-20 Cell Analyzer (BD Biosciences, California, U.S.A.).
Software	Data acquisition and analysis was performed using FACsDiva versions 8.0.1 and 9.0.1 (BD Biosciences) and FlowLogic software version 8.6 (Inivai).
Cell population abundance	Cell population abundance and purity was assessed via flow cytometry detection of endogenous fluorescence reporters that specifically mark kidney cell populations of interest within kidney organoids, or by staining with directly conjugated fluorescent antibody to identify nephron epithelium. Kidney cell type abundances are stated throughout the manuscript, ranging from ~4% - ~88% depending on the cell type.
Gating strategy	FSC/SSC analysis was initially performed to identify individual live cells and exclude cell debris and clusters of two or more cells. Dissociated control organoids (that did not contain a fluorescent reporter) were used as a gating control for identifying cells expressing the reporter gene of interest (EGFP, YFP and/or mCherry). Only the live, single cell population was analysed for reporter gene expression.

- [x] Tick this box to confirm that a figure exemplifying the gating strategy is provided in the Supplementary Information.
